# Supplementary material for: Experimental evidence supporting a global melt layer at the base of the Earth’s upper mantle
Source: Nat Commun. 2017 Dec 19;8:2186. doi: 10.1038/s41467-017-02275-9 (PMC5736617; doi:10.1038/s41467-017-02275-9)
Supplement: Supplementary file 1 — Supplementary Information [file 41467_2017_2275_MOESM1_ESM.pdf]

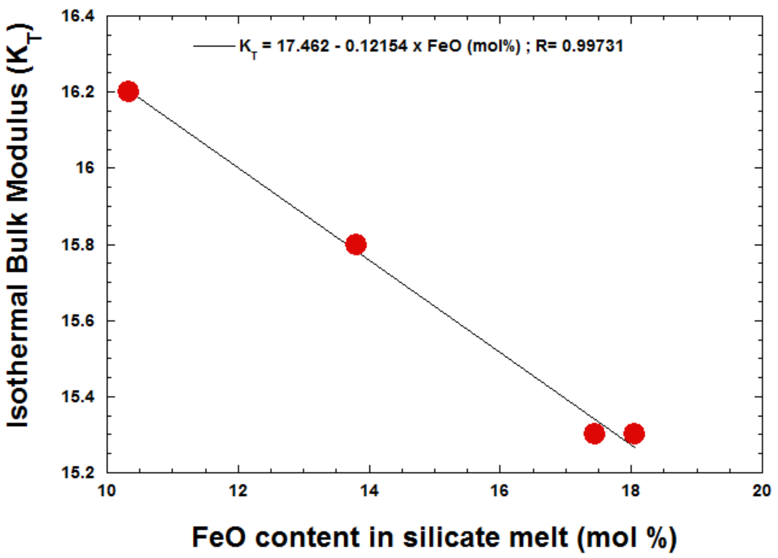

Supplementary Figure 1. Isothermal bulk modulus (K_T) as a function of the FeO content (mol. %).

	Peridotite	2 % melt	7 % melt	25 % melt
SiO_2	44.48	39.74	45.59	45.09
TiO_2	0.16	0.00	0.93	0.52
Al_2O_3	3.59	2.55	10.09	11.20
Cr_2O_3	0.30	0.00	0.00	0.00
FeO	8.10	21.23	11.34	6.48
MnO	0.12	0.00	0.05	0.00
MgO	39.22	2.62	10.69	16.01
CaO	3.44	8.95	16.63	14.75
Na_2O	0.30	0.07	1.52	1.63
K_2O	0.02	0.13	0.16	0.04
H_2O	0.02	12.00	4.20	0.30
**Total**	**99.75**	**87.30**	**101.22**	**96.01**
**φ (°)**		**5.98**	**7.01**	**8.50**

Supplementary Table 1. Chemical compositions of the initial peridotite mixture and the resulting hydrous silicate melts. The median dihedral angles (φ) are shown for each melt fraction with ±2° uncertinity.
